# Metabolomics in microbiology: interrogating microbial physiology and interactions

**DOI:** 10.1128/mbio.01913-26

**Published:** 2026-10-05

**Authors:** Mónica Monge-Loría, Andrew C. McAvoy, Neha Garg

**Affiliations:** 1 School of Chemistry and Biochemistry, Georgia Institute of Technology 1372, Atlanta, Georgia, USA; 2 Center for Microbial Dynamics and Infection, Georgia Institute of Technology 1372, Atlanta, Georgia, USA; Georgia Institute of Technology, Atlanta, Georgia, USA

**Keywords:** metabolomics, microbe-host interactions, microbe-microbe interactions, chemical microbiology

## Abstract

Metabolomics provides a direct window into microbial function by capturing the small molecules that reflect cellular activity and microbial interactions. Here, we highlight its diverse applications for microbiologists across both *in vitro* and *in vivo* models under dynamic biological and nutritional conditions, including variations in community complexity, medium composition, and exposure to exogenous compounds such as antibiotics. Through representative case studies, we demonstrate how metabolomics provides critical insights into microbial physiology and inter-organismal interactions. We also showcase examples of multiomics integration, in which metabolomics is combined with complementary approaches to enhance data interpretation and strengthen biological conclusions. Finally, we discuss key challenges in metabolomics data analysis and highlight emerging strategies to overcome these limitations.

## INTRODUCTION

The version of the central dogma of molecular biology as popularized by James Watson states that “DNA → RNA → protein” (1). This oversimplified framework has since been challenged by the discovery of biological phenomena such as reverse transcription, RNA replication, post-translational modification, and epigenetic regulation. Notably, the central dogma fails to consider the critical role that small molecules play at all levels of biological complexity. Small molecules are able to modulate all steps in the flux of genetic information through mechanisms such as feedback inhibition and cell signaling, leading to a revision of the narrow view of metabolites as simple downstream products of gene and protein expression (2). The inclusion of small molecules in the central dogma was first suggested by Schreiber (3), almost 50 years after the conception of the central dogma. The full set of small molecules within a biological sample, referred to as the metabolome, includes endogenous metabolites produced by the organism as well as exogenous chemicals from the surrounding environment. Microbial metabolism consists of primary metabolites that are necessary for growth, and secondary or specialized metabolites that mediate interactions with the extracellular environment (4). In the postgenomic era, metabolomic profiling is routinely performed to obtain a global snapshot of the physiological state of an organism under a specified set of conditions. As a direct reflection of biochemical activity, the metabolome is influenced by an interplay between a cell’s genome and its external environment. Hence, metabolomic analysis is frequently used by microbiologists as a useful proxy for the molecular phenotype.

Herein, we first describe how metabolomics can be applied within the context of microbiological investigations and present examples for several use cases. We next describe analytical techniques that are commonly used for metabolomic analysis. Finally, we conclude with a discussion on the outstanding challenges in the field and prospective strategies for addressing these challenges. Ultimately, the goal of this article is to highlight the potential of metabolomics in microbiology and introduce these methods to microbiologists who are interested in implementing it in their own research.

## APPLICATIONS OF METABOLOMICS IN MICROBIAL RESEARCH

**Fig 1:**
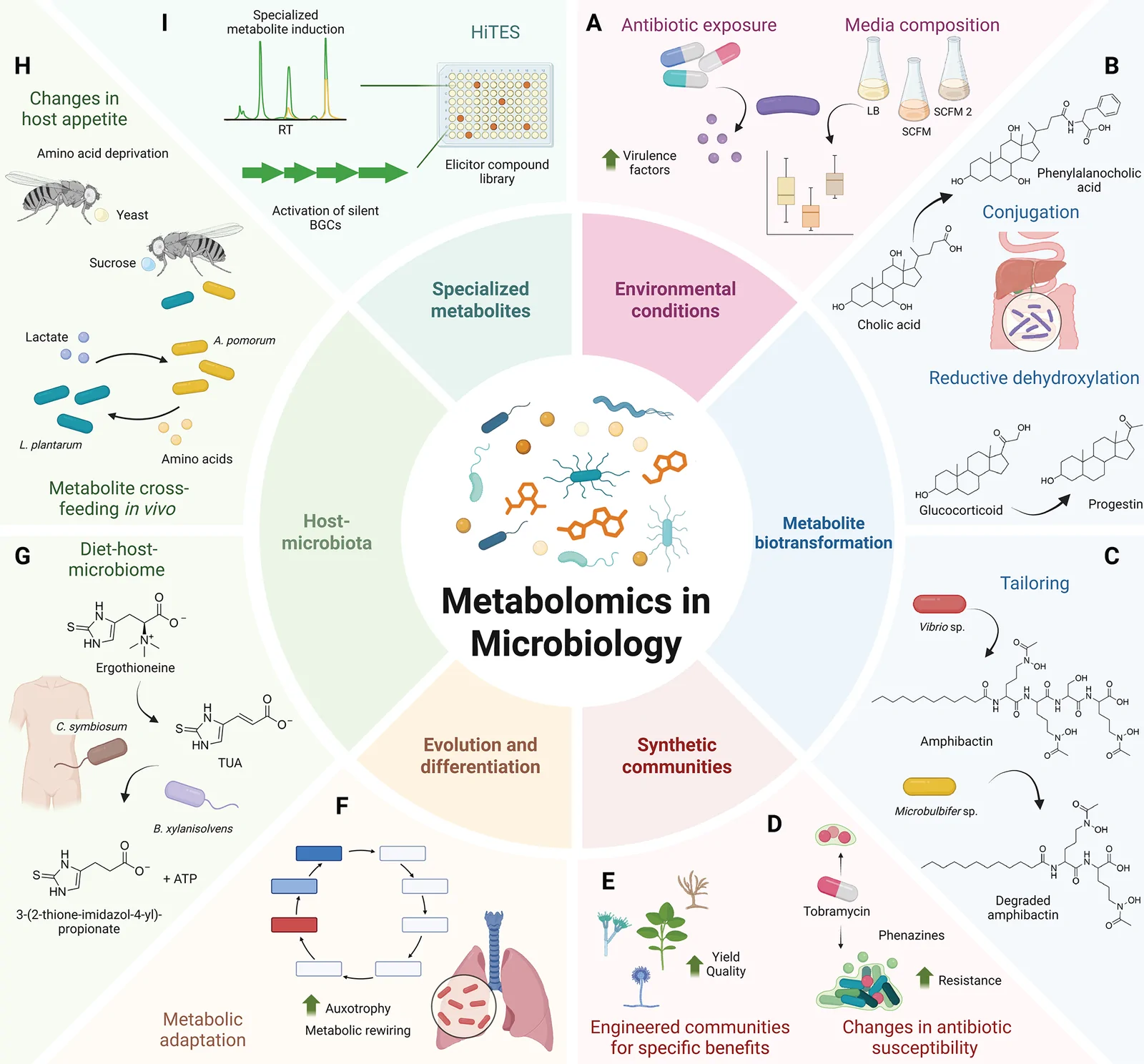
Applications of metabolomics in microbiology research. Metabolomics provides a versatile platform for investigating microbial physiology, metabolism, and interactions across diverse biological systems. Representative applications include (**A**) studying microbial responses to environmental perturbations, investigating microbial metabolite biotransformations through (**B**) conjugation, reductive dehydroxylation, and (**C**) tailoring, engineering synthetic microbial communities to understand community function and improve traits such as (**D**) beneficial host interactions and (**E**) antibiotic susceptibility, (**F**) probing microbial metabolic adaptation to the host environment, identifying host-microbiota (**G**) interactions and (**H**) resulting phenotypic changes, and (**I**) characterizing specialized metabolite biosynthesis through high-throughput elicitor screening (HiTES) and genome-guided data mining. Together, these applications illustrate the broad utility of metabolomics for generating mechanistic insights into microbial biology and chemical communication. The green upward arrow denotes the increase.

One of the earliest applications of mass spectrometry (MS) in microbiology was the identification and classification of bacteria based on characteristic mass spectral fingerprints, first demonstrated in 1975 (5). This approach was subsequently transformed by the development of matrix-assisted laser desorption ionization coupled to time-of-flight mass spectrometry (MALDI-TOF MS), which is now routinely used for the rapid identification of bacterial pathogens in clinical microbiology laboratories (6, 7). In recent years, MS-based metabolomics has become an indispensable tool in microbiology, enabling researchers to characterize microbial responses to environmental and chemical perturbations. Experimentally tractable systems, including synthetic microbial communities and defined animal models such as gnotobiotic (germ-free) and specific pathogen-free (SPF) mice, are particularly well suited for metabolomic studies because they enable controlled perturbations and facilitate mechanistic investigation of microbial physiology and host-microbe interactions in defined biological and chemical contexts. Using metabolomics, researchers can discover biomarkers and bioactive compounds, elucidate the modes of action of antimicrobial agents, assign functions to previously uncharacterized genes, and generate mechanistic hypotheses for host-microbe interactions, among numerous other applications (Fig. 1). Representative examples illustrating these applications are discussed henceforth.

### Probing microbial responses to environmental perturbations through metabolomics

Microbial metabolism is highly influenced by the surrounding chemical and physical environment, making it essential to perform metabolomic studies under conditions that closely reflect the biological system of interest. One notable example is cystic fibrosis (CF), a genetic disease characterized by chronic respiratory infections (8, 9), where the airway presents a unique biochemical milieu. To better investigate pathogen physiology under clinically relevant conditions, several synthetic sputum media have been developed to mimic the chemical and physical properties of CF airway secretions (10–12). Metabolomic profiling reveals that synthetic cystic fibrosis sputum media (SCFM and SCFM2) induce extensive metabolic remodeling in *Pseudomonas aeruginosa* and *Burkholderia cenocepacia* relative to standard laboratory media such as Luria-Bertani (LB), altering the production of metabolites associated with virulence and pathogenesis (Fig. 1A) (13–15). Beyond nutrient composition, exposure to sublethal concentrations of clinically relevant antibiotics further reshapes the metabolome by inducing the production of specialized metabolites and small-molecule virulence factors. Moreover, strain-specific microbial phenotypes, such as variation in pigment production, underscore the combined influence of genetic background and environmental cues on microbial metabolism and virulence (16).

In addition to mapping medium-dependent metabolic shifts, metabolomics can inform the rational design of physiologically relevant culture media. For example, metabolomic profiling of *Trypanosoma brucei* identified the metabolites utilized by the parasite during sustenance in blood, providing the foundation for developing blood-mimicking media that support robust *in vitro* growth (17). Similar approaches have been applied to model diverse environments, including soil (18, 19), foods (20), as well as plant and insect hosts (21). By defining the metabolic composition of native niches, metabolomics enables the development of culture media that recapitulate *in vivo* conditions, facilitating the cultivation of otherwise difficult-to-culture microorganisms and improving the physiological relevance of experimental findings.

### Discovery of microbiome-derived biotransformations

Owing to advances in data analysis tools in metabolomics, metabolite modification by the microbiome has gained substantial attention. For instance, a metabolomics pipeline developed by Balskus and colleagues revealed differential detection of acetylated polyamines in bloodstream infections, production of which was then linked to a bacterial acetyltransferase enzyme (22). Functional characterization showed that acetylation of polyamines is not just metabolic noise driven by enzyme promiscuity but actively supports pathogen survival and proliferation via modulation of membrane properties that contribute to antibiotic tolerance. Indeed, inhibition of this enzyme re-sensitized multi-drug-resistant pathogens to various antibiotics, likely through the modulation of membrane charge and structure (22). This work highlights the potential of metabolomics to identify disease-associated metabolites, generate mechanistic hypotheses, and uncover bacterial enzymes such as SpeG as therapeutic targets for restoring antibiotic susceptibility in multidrug-resistant pathogens.

The discovery of microbial biotransformations of steroidal compounds further illustrates the power of metabolomics to uncover previously unrecognized metabolic activities. The production of novel bile acid conjugates (e.g., phenylalanine, tyrosine, and leucine conjugates of cholic acid) that are not produced by the host, but by the gut microbiome, has revolutionized the application of reverse metabolomics in the discovery of new chemistry at the interface of host-microbiome interactions (Fig. 1B) (23–25). These methods have revealed an unexpectedly vast and diverse landscape of microbially catalyzed bile acid conjugations, so far spanning the thousands (25, 26). These secondary bile acids can impact host phenotype, function as signaling molecules and as metabolic and immune modulators, and can restructure the gut microbiota (27–29). Similarly, the gut microbiota can also transform glucocorticoids into progestins (Fig. 1B), mediated by bacterial H_2_ production (30). This biotransformation effectively changes the functional class of steroids from immune modulation to sex hormones and neuromodulation, therefore exemplifying how microbial biotransformations can impact host physiology. Notably, while mass spectrometry enabled the detection of microbiome-derived steroid metabolites, the use of SPF mice was essential to demonstrate that these transformations occur in a microbiota-dependent manner *in vivo*. Thus, a combination of advanced animal models and mass spectrometry is critical for establishing a direct functional and causal link between bacterial metabolism and host physiology.

Apart from the biotransformation of host-derived metabolites, modifications that stem from microbe-microbe interactions can also be identified through metabolomics. For example, the bacterially produced compound fragin was shown to be methylated by the secondary pathogen *Aspergillus fumigatus* via the application of advanced cheminformatics methods for the analysis of mass spectrometry data (31). Fragin is a metal-chelating metabolite that exhibits antifungal activity through sequestration of copper, thereby limiting fungal growth. Thus, by methylating one of the oxygens involved in copper binding, *Aspergillus* hampers the antifungal activity of this compound. Similar methodologies enabled the description of pyochelin methylation by *S. aureus* and the first proteolytic degradation of amphibactin siderophores, produced by *Vibrio* sp., by marine *Microbulbifer* spp. bacteria (Fig. 1C) (32, 33). As with fragin methylation, these biotransformations are proposed to disrupt the siderophores’ function, benefiting the tailoring microbe.

Previously uncharacterized pathways of antibiotic transformation and inactivation can give rise to strain-specific antibiotic resistance that is not apparent from genome analysis alone. Consequently, mass spectrometry-based metabolomics is required to identify these cryptic resistance mechanisms. Garg and co-workers developed a substructure-guided stable isotope-labeling approach that uncovered a previously unrecognized, strain-specific mechanism of trimethoprim inactivation linked to pigment biosynthesis (16). An extensive analysis of drug microbial metabolism revealed structural features that are prone to biotransformation, as well as common reactions carried out by gut microbiota (34). This study revealed the proclivity of steroids, ketones, and nitro group-containing drugs to microbial biotransformation.

A related example is the metabolism of catechol-*O*-methyltransferase inhibitor (COMT-I) drugs, which brought forward the nuances of drug-drug interactions as mediated through microbial metabolism. The COMT-I inhibitors are commonly co-administered during Parkinson’s disease treatment to inhibit peripheral metabolism of L-DOPA, the standard therapeutic agent. Metabolomics analyses have revealed the biotransformation of COMT-I drugs by gut microbiota, resulting in selective antibacterial activity (35). Therefore, COMT-I biotransformation reshapes the microbial community, resulting in unintended expansion of *Enterococcus*. In turn, these compositional shifts alter microbial metabolism of L-DOPA, ultimately reducing its therapeutic efficacy (35). Thus, co-administration ultimately has negative effects, as microbial metabolism of COMT-I drugs counteracts their intended purpose. These findings highlight the application of metabolomics analyses to delineate unintended drug biotransformations and their downstream ecological effects, suggesting that these analyses are of importance to inform the design of more effective therapeutic strategies and drug combinations. Finally, these examples illustrate how microbial metabolism of bioactive compounds can influence community composition, highlighting the role of microbe-drug-microbe interactions.

### Metabolomics studies of synthetic communities

Synthetic communities comprise defined microbial consortia, thoughtfully curated for targeted functional outcomes (36). Within these systems, interspecies interactions can drive compound biotransformations and facilitate the cooperative biosynthesis of specialized metabolites, as previously covered. Additionally, microbes in synthetic communities can reciprocally induce each other’s secondary metabolism; consequently, integration of synthetic communities and metabolomic profiling has resulted in the discovery of new bioactive compounds (37, 38).

In addition to changes in the secondary metabolome, primary metabolic pathways work in concert within synthetic communities, leading to microbes coupling and changing their carbon and nitrogen metabolism. Typical metabolomic profiling of a single microbe is therefore not representative of its behavior in a mixed community, as encountered in the natural environment. However, assigning metabolites to their producing organisms and disentangling metabolic interactions in mixed communities remains a challenging feat. Synthetic microbial communities provide a tractable solution by enabling controlled, stepwise increases in community complexity while preserving the ability to link metabolic phenotypes to individual community members. Metabolomics provides a useful approach for resolving metabolic division of labor within microbial communities, enabling the rational engineering and optimization of community composition (39). More recently, metabolomic data have been integrated with genome-scale metabolic flux models and other omics data sets to decipher cooperative and competitive interactions in synthetic communities, revealing how metabolic roles shift in response to changes in community composition (40–45).

Polymicrobial models also facilitate the identification of community-dependent changes in antimicrobial susceptibility that are mediated by microbial metabolites. For instance, the sensitivity of cystic fibrosis-associated pathogens to the clinically relevant antibiotic tobramycin is highly dependent on community composition (46). This increased resistance is driven by phenazines that are redox-active, specialized metabolites whose production is induced in mixed communities (Fig. 1D) (46, 47). Synthetic communities can also aid in the identification of microbial drug biotransformation, in which community members biochemically modify xenobiotics, thereby altering their physicochemical and pharmacological properties (48). One can articulate that such biotransformation can alter the ecological effects of the drug, ultimately reshaping the composition and function of the microbial community present.

Beyond elucidating microbial interactions, synthetic communities provide a platform for engineering microbiomes with desirable functions. By systematically manipulating community composition and monitoring metabolic outputs, metabolomics can identify community traits that promote specific outcomes. For example, synthetic plant-associated communities have been engineered to enhance plant growth, suppress phytopathogens, and improve resilience to environmental stress (49–51). Fungal synthetic communities have also been used to enhance the yield and quality of medicinal plant biomass by modulating host metabolism (Fig. 1E) (52). Metabolomic analyses revealed that changes in community composition differentially affected primary and specialized metabolism, altering central metabolic pathways such as the tricarboxylic acid (TCA) cycle as well as the accumulation of bioactive specialized metabolites. In this study, inoculation of closely related fungal species resulted in cooperation rather than competition, whereas introduction of a different fungal family resulted in heightened competition, inducing stress on the plant and increasing production of active metabolites (52). These synthetic communities can therefore be used to increase crop productivity and resilience. In addition to plant microbiomes, metabolomic analyses of synthetic gut communities have revealed beneficial effects of Stickland-fermenting gut bacteria in the suppression of *Clostridioides difficile* through competition for proline (53). Altogether, these examples demonstrate the important role of metabolomics in the identification of beneficial traits from synthetic communities, rational optimization of community composition, and the characterization of the metabolic rewiring that underlies desirable phenotypes.

### Probing evolution and differentiation through metabolomics

Metabolomics provides a direct readout of the biochemical changes that accompany microbial evolution, adaptation, and differentiation. Thus, metabolomics enables the identification of metabolic traits that emerge in response to new environmental pressures. Because metabolic phenotypes integrate the effects of genetic and regulatory changes, comparative metabolomics can reveal adaptive strategies that are not readily apparent from genomic analyses alone. For example, *P. aeruginosa* has been studied as a model for adaptation within the host in the disease CF, identifying metabolic changes during adaptation from the environment to the airway niche. Comparative metabolomics of *P. aeruginosa* clinical isolates revealed metabolic specialization concurrent with evolution within the host. Metabolism of amino acids, organic acids, and sugars varied between naive and adapted isolates, showing nutritional adaptation and changes in metabolic flux (54). As *P. aeruginosa* became increasingly adapted to the CF airway, metabolomic analyses also revealed the progressive loss of non-essential metabolic functions such as reduced carbon source versatility and the emergence of auxotrophy reflecting specialization to the nutrient-rich sputum environment (Fig. 1F) (54). Metabolomics has likewise provided key insights into adaptive evolution in the *Escherichia coli* long-term evolution experiment (LTEE), spanning more than 30 years. Comparative metabolomic analyses of evolved lineages revealed widespread remodeling of central carbon metabolism, amino acid metabolism, and nicotinamide adenine dinucleotide (NAD) biosynthesis (55). Additionally, changes in metabolic pathways are linked to increased growth rates, accelerating energy demands. However, these changes were accompanied by reduced metabolic flexibility, as evolved lineages exhibited a diminished capacity to utilize diverse carbon sources (55).

Another form of adaptation that can be mapped using metabolomics is antibiotic resistance. Exposure to antibiotics induces extensive metabolic reprogramming that enables bacterial survival prior to the acquisition of stable genetic resistance, thereby increasing the likelihood that resistance-conferring mutations will arise. To identify metabolic signatures associated with this pre-genetic adaptive state, metabolomics-driven intervention antibiotic design (MDAD) was developed. This strategy provides metabolic biomarkers that predict the emergence of resistance (56). Across diverse bacterial species and antibiotic classes, perturbations in primary metabolism, particularly amino acid and lipid metabolism, represent conserved hallmarks of this adaptive transition (57–59). Metabolic specialization can also influence pathogenicity to favor long-term chronic infections by rewiring primary metabolism and pyruvate secretion (60). Together, these studies demonstrate that metabolomics can identify early metabolic predictors of antibiotic resistance and reveal adaptive mechanisms that influence treatment outcomes.

### Probing host-microbiota interactions through metabolomics

Microorganisms establish complex relationships with their hosts through small molecule exchange that shapes the physiology of both partners. By providing a comprehensive snapshot of these molecular interactions, metabolomics has become an indispensable tool for elucidating the biochemical mechanisms underlying host-microbiota relationships. Although several examples discussed in previous sections involved host-associated systems to investigate microbial physiology and environmental adaptation, this section focuses specifically on direct host-microbiota interactions. One example is the emerging role of the gut microbiota in regulating ergothioneine metabolism and redox homeostasis. Ergothioneine, a sulfur-containing derivative of histidine (61), is obtained by the host through the diet and can be imported by some species of gut bacteria (62). Bacterial uptake of ergothioneine enhances resistance to oxidative stress (63), resulting in a competitive advantage that can influence microbial community composition. Indeed, the prevalence of ergothioneine transport genes in host-associated bacteria from diverse phyla suggests a role in adaptation to the host environment (62, 64). Metabolomic analyses further revealed that gut bacteria can biotransform ergothioneine into downstream metabolites, including trimethylamine (TMA) and trimethylamine *N*-oxide (TMAO), linking microbial metabolism to host health through both depletion of a beneficial antioxidant and production of metabolites associated with disease (62). Notably, metabolomics was essential for uncovering this interaction because ergothioneine is not synthesized *de novo* by the bacteria, and the transporters were unknown.

In addition to mediating host-microbiota interactions, ergothioneine also shapes bacterial interspecies dynamics through metabolite cross-feeding. Conversion of ergothioneine to TMA by gut bacteria such as *Clostridium symbiosum* produces thiourocanate (TUA). TUA is, in turn, utilized by other symbionts, such as *Bacteroides xylanisolvens*, for energy metabolism and converted into 3-(2-thione-imidazol-4-yl)-propionate (Fig. 1G), serving as an alternative electron acceptor under anaerobic conditions (65). This metabolic cascade illustrates how the transformation of a single dietary metabolite can drive metabolic cooperation between microbial species while also influencing host physiology. More broadly, these findings highlight the power of metabolomics to resolve metabolic cross-feeding and trace the flow of metabolites across complex host-microbiota networks, exemplifying the intimate connections among diet, the microbiome, and host metabolism.

Metabolomics has also provided important insights into the role of the gut microbiome in cardiovascular disease (66). Despite interindividual variation arising from genetic background, diet, and other cardiovascular risk factors, metabolomic analyses have identified conserved metabolic alterations associated with disease, particularly dysregulation of lipid metabolism (66). For example, the gut microbiota influences key cardiovascular biomarkers, including plasma triglycerides, cholesterol, and blood pressure (67). Human cohort studies revealed that lower circulating cholesterol levels are associated with increased fecal concentrations of coprostanol, a microbial metabolite of cholesterol. Metabolomic and microbiome analyses identified specific gut bacteria capable of converting cholesterol into coprostanol and other oxysterols, thereby promoting fecal excretion of cholesterol and reducing circulating cholesterol levels in the host (67).

The gut bacteria can also modulate host diet preference and reproductive fitness in the host, as shown in *Drosophila melanogaster* (Fig. 1H) (68). Commensal bacteria, such as *Acetobacter pomorum* and *Lactobacillus plantarum,* buffer the dietary amino acid deficiency, thereby preventing the reductions in fecundity and the shifts in feeding preference between sucrose- and yeast-rich diets that accompany nutrient deprivation (68). Metabolomic analyses revealed that these effects are mediated by lactate and amino acid cross-feeding between the bacterial symbionts (Fig. 1H), which, in turn, drives the production of specialized metabolites that influence host physiology and behavior (69). These examples underscore the importance of metabolomics to uncover the molecular mechanisms through which microbial metabolism shapes host nutrition, reproduction, and other complex phenotypes.

### Metabolomics for the discovery of specialized metabolites

Microorganisms produce a vast repertoire of specialized metabolites that function as key mediators of microbial ecology and host-microbe interactions, influencing processes such as interspecies competition, immune modulation, host colonization, and virulence (70–72). These molecules also constitute a rich reservoir of bioactive compounds (73). To facilitate their discovery, untargeted (74) metabolite profiling can now be easily integrated with traditional bioactivity-guided discovery workflows. Toward this goal, several expertise-agnostic and user-friendly computational tools have been developed that combine bioactivity data with molecular networking to prioritize bioactive metabolites (75). These integrated approaches have facilitated the discovery of microbial natural products with diverse biological activities, including antibacterial (76) and anticancer activity (77). Beyond integrating metabolomics with bioactivity-guided discovery, multi-omic approaches that combine metabolomics with genomics, transcriptomics, and proteomics are increasingly being used to accelerate specialized metabolite discovery (78–80). By linking metabolite production to biosynthetic genes, gene expression, and protein abundance, these complementary data sets enable high-throughput prioritization of novel metabolites while providing mechanistic insight into their biosynthesis and regulation. Recent studies using integrated metabolomic and genomic workflows have enabled the discovery of the metallophore family of delftichelins from *Delftia deserti* (81) and the antibacterial embleyamycins (82), highlighting the power of combining complementary data sets to uncover specialized metabolites that mediate microbial interactions and facilitate environmental adaptation.

Metabolomics can also be guided by prior knowledge of target chemistries to enable the focused discovery of specialized metabolites. Incorporation of chemical tags or stable isotopes facilitates selective detection of chemistry of interest. One such computational tool is mass spectrometry query language (MassQL), which enables users to search mass spectrometry data sets for metabolites with defined spectral features, such as characteristic fragment ions or neutral losses (27). The recent integration of AI-assisted tools that translate natural language into MassQL syntax has further lowered the barrier to query development and broadened accessibility to microbiologists who may not have prior coding experience. MassQL has been used to systematically identify diverse classes of specialized metabolites, including homoserine lactones, halogenated metabolites, organophosphates, and perfluorinated sulfonates. These targeted workflows have recently enabled the discovery of proline-containing cyclic peptides (83) and siderophores (84) from microbial extracts.

Chemical reactivity can also be exploited to selectively enrich, detect, and identify specific classes of specialized metabolites. For example, selective derivatization was used to detect a quorum-sensing (QS) autoinducer mimic containing a diketone functionality (85). Derivatization enhanced detection by both UV-based isolation and mass spectrometry, generating a diagnostic signature that enabled targeted metabolite tracking. Remarkably, this metabolite is produced by mammalian cells and mimics the bacterial autoinducer AI-2, activating quorum-sensing pathways that regulate microbial gene expression, physiology, and community behavior (85, 86). Reactivity-guided metabolomics has also been applied to capture diazo-containing specialized metabolites, where derivatization of the highly reactive diazo group stabilized these metabolites and improved their detection (87). The diazo-containing metabolites possess diverse biological activities such as antibiotic and anticancer properties. Thus, they are considered high-priority targets for specialized detection strategies.

A major challenge in specialized metabolite discovery is that many bacterial biosynthetic gene clusters (BGCs) remain transcriptionally silent under standard laboratory conditions, limiting their discovery by metabolomics. To overcome this barrier, HiTES was developed as a strategy in which libraries of small molecules are used to activate silent BGCs without requiring genetic manipulation (Fig. 1I) (88). Metabolome changes are subsequently monitored by mass spectrometry to identify induced metabolites. This approach has enabled the discovery of numerous specialized metabolites including antibiotics (89, 90), antiproliferative metabolites (91), and diazeniumdiolate siderophores (92) from both well-known and understudied microorganisms. A HiTES approach surveying hundreds of elicitors aided in the identification of a new family of lipopeptides from *Bacteroides dorei* named doreamides. These compounds exhibit pro-inflammatory activity in macrophages and induce the production of host antimicrobial compounds, reshaping the microbiome (93).

Secondary metabolite production can also be elicited through microbial co-cultivation, as previously described for synthetic communities. In addition to interspecies microbial competition, exposure to host immune cells serves as a critical environmental cue that stimulates microbial secondary metabolism and alters chemical output. For instance, culturing the pathogenic fungus *A. fumigatus* with macrophages induces the production of fumigatinolactone (94). This compound is produced through a survival interaction mechanism, via the non-enzymatic coupling of fumigatin and fumarylazlactone, which are induced by macrophage NO production and iron sequestration, respectively (94). A similar approach enabled the elucidation of the cytotoxic and anti-inflammatory uniformides A and B, whose production by *Nocardia uniformis* is induced in coculture with macrophage-like cells (95). These studies demonstrate how targeted induction of specialized metabolism, coupled with comparative metabolomics, provides an activity-agnostic strategy for uncovering cryptic metabolites that would otherwise remain undetected.

## ANALYTICAL TECHNIQUES FOR METABOLOMIC PROFILING

**Fig 2:**
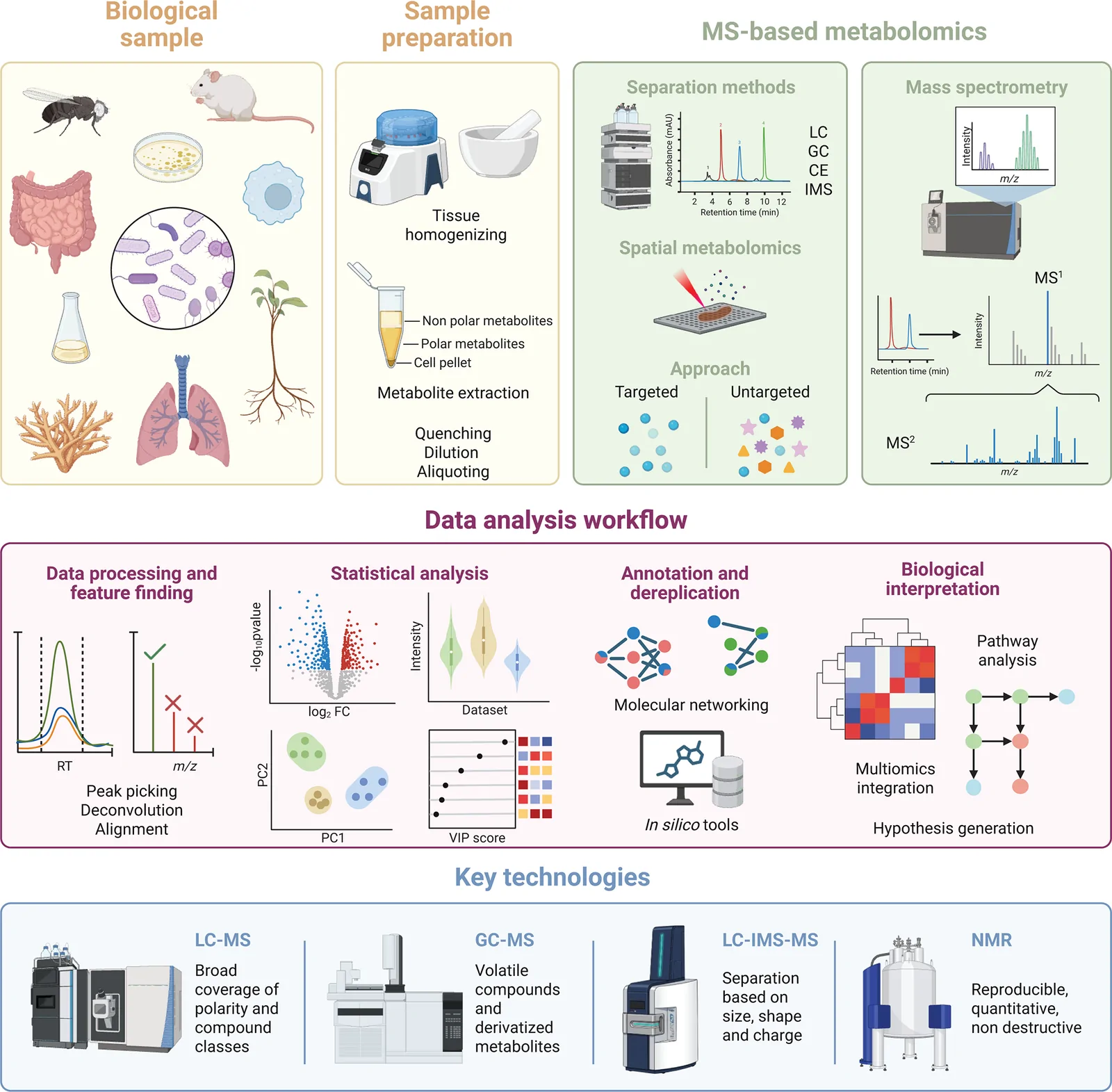
Analytical workflows and mass spectrometry-based methodologies for metabolomic profiling. Metabolites are extracted from diverse biological sources for metabolomics data acquisition. Mass spectrometry is often coupled with separation methods to increase metabolite coverage; alternatively, mass spectrometry imaging can be performed to retain spatial context on metabolite distribution. Depending on the study objectives, data can be acquired via targeted or untargeted profiling approaches. Following acquisition, precursor m/z and fragmentation data are subjected to metabolomics data analysis workflows for metabolite annotation and biological interpretation. Key analytical technologies for metabolomics are highlighted.

Recent advances in analytical instrumentation have greatly expanded the scope and sensitivity of metabolomic profiling. Metabolomic data are primarily acquired using nuclear magnetic resonance (NMR) spectroscopy or mass spectrometry (MS) (Fig. 2). Although both platforms provide valuable and complementary information, MS has become the predominant technology for global metabolomics because of its superior sensitivity, selectivity, and metabolome coverage, enabling the detection of thousands of metabolites from complex biological samples (96). In MS-based metabolomics, ionized metabolites are detected according to their mass-to-charge ratio (m/z). Structural information is obtained through tandem mass spectrometry (MS/MS or MS^2^), in which selected precursor ions are fragmented, and the resulting product ions are analyzed to generate characteristic fragmentation spectra. These spectra provide important structural information that can be used for metabolite annotation, identification, and dereplication. In some applications, product ions can undergo additional rounds of fragmentation (MSⁿ) to provide further structural detail. MS-based metabolomics can be performed in either an untargeted or targeted manner. Untargeted metabolomics aims to comprehensively profile the metabolites present in a sample, whereas targeted metabolomics focuses on the sensitive and quantitative analysis of predefined metabolites. Readers interested in a more comprehensive discussion of MS instrumentation and metabolomic workflows are referred to several excellent reviews on this topic (97–99).

Conventional metabolomics analyses are performed on bulk biological samples, in which metabolites are extracted using solvent systems tailored to the chemical properties of the target metabolome. Owing to the complexity of these extracts, mass spectrometry is commonly coupled with separation techniques to improve metabolome coverage and depth, reduce ion suppression, and increase confidence in metabolite identification. Common hyphenated platforms include liquid chromatography (LC-MS), gas chromatography (GC-MS), capillary electrophoresis (CE-MS), and ion mobility spectrometry (IMS). The choice of separation method depends on the physicochemical properties of the metabolites of interest. For example, GC-MS is well suited for volatile compounds and primary metabolites following chemical derivatization (100, 101). CE-MS provides excellent separation of highly polar and charged metabolites (102), while LC-MS offers broad applicability across diverse metabolite classes (103). On the other hand, IMS separates ions in the gas phase according to their size, shape, and charge, enabling structural characterization and the resolution of isomeric compounds (104). Together, these hyphenated methods provide additional information beyond *m/z*, including retention time (RT) for LC and GC, migration time (t_m_) for CE, and collision cross section (CCS) for IMS. These orthogonal molecular descriptors substantially improve metabolite annotation and increase confidence in compound identification.

Although conventional metabolomics provides a comprehensive snapshot of the biochemical state of a biological sample, it lacks spatial information that is often critical for understanding metabolite function in complex tissues and polymicrobial communities. Mass spectrometry imaging (MSI) allows the visualization of the spatial distribution of metabolites directly within a biological sample (105). Unlike fluorescence- or antibody-based imaging approaches, MSI does not require molecular labeling and enables the simultaneous detection of thousands of metabolites in a single experiment. During MSI acquisition, a mass spectrum is collected at each pixel across a predefined x-y grid, and ion intensity maps are reconstructed to visualize the spatial localization of individual metabolites. Coupling chemical identity with spatial context provides valuable insights into metabolite function, microbial interactions, and the biosynthetic origins of small molecules.

MSI has proven particularly powerful for investigating microbial interactions in Petri dishes. For example, an MSI-based workflow was used to characterize metabolic interactions between *P. aeruginosa* and *A. fumigatus* grown in coculture on agar (106). *A. fumigatus* lacks the biosynthetic machinery to produce phenazines, but phenazine metabolites were found to be localized around the fungal colony. Subsequent analyses demonstrated that phenazine-1-carboxylic acid produced by *P. aeruginosa* was biotransformed by *A. fumigatus* into multiple structural analogs through decarboxylation, hydroxylation, methylation, and sulfonation. This work illustrates how MSI can reveal spatially resolved metabolic transformations that may be difficult to identify using bulk metabolomic analyses alone. More broadly, the ability of MSI to combine chemical specificity with spatial resolution has made it an indispensable tool for metabolically mapping complex microbial ecosystems, including the human skin microbiome, symbiotic bacterial communities associated with shipworm gills, and synthetic microbial communities in soil-mimicking matrices (107–109). These approaches enable spatial metabolite patterns to be linked to microbial localization and community organization, providing insights into metabolic interactions that may be obscured by bulk metabolomic analyses.

Although this review has focused on applications of metabolomics in microbiological research, metabolomics represents only one component of the omics landscape. Together with genomics, transcriptomics, proteomics, and other systems-level approaches, metabolomics contributes complementary information enabling a comprehensive understanding of biological systems. While each omics technology can independently address specific biological questions, integrating multiple data sets has become increasingly common because it enables the direct linkage of genotype, gene expression, protein abundance, and metabolic phenotype. The usefulness of multi-omics integration is illustrated by a recent investigation on *S. aureus* survival in blood serum. Transcriptomic, proteomic, and metabolomic analyses revealed adaptive responses to this niche, including changes in carbohydrate, lipid, and nucleotide metabolism, as well as dysregulation in iron uptake and oxidative stress resistance pathways (110). This study highlights how multi-omics integration can build mechanistic hypotheses underlying host-associated microbial survival and adaptation, paving the way for targeted therapeutic strategies against bacterial pathogens.

An additional multi-omics study recently investigated the antitumor activity of *Lycium barbarum* polysaccharides (LBP) (111). By combining metabolomics with transcriptomics, the authors demonstrated that LBP suppresses cancer cell proliferation by inducing ferroptosis. Transcriptomic analyses revealed dysregulation of iron homeostasis and oxidative stress pathways, whereas metabolomic profiling identified perturbations in amino acid, glutathione, and lipid metabolism that collectively supported increased reactive oxygen species production, lipid peroxidation, and ferroptotic cell death. Although this example lies outside microbiology, it further illustrates how integrating complementary omics data sets can reveal molecular mechanisms that would be difficult to resolve using a single analytical approach.

## CHALLENGES IN METABOLOMICS

Metabolomics has become an indispensable tool for studying microbial processes and holds great promise for advancing microbial research; however, several technical and biological challenges continue to limit its wider implementation among microbiologists. First, identifying the entire set of intra- and extra-cellular small molecules in a sample is technically difficult, and there is no universal MS approach that can realistically achieve this goal in a single run. Unlike genomes, transcriptomes, or proteomes, microbial metabolomes are not finite and cannot be readily predicted from genomic information alone. Furthermore, metabolite coverage is influenced by experimental design. Sample preparation, extraction solvents, chromatographic separation, ionization mode, and mass spectrometry acquisition parameters each introduce biases toward classes of metabolites detected, while some compounds remain refractory to MS detection regardless of the analytical platform employed. As a result, even untargeted metabolomics experiments provide only a partial view of the metabolome. To maximize coverage, researchers often combine complementary extraction procedures, chromatographic methods, ionization modes, and analytical platforms, although these approaches increase experimental complexity and cost.

Another challenge arises, given the dynamic nature of metabolites. Metabolites are in constant flux, and microorganisms can present markedly different metabolomes at various growth stages, making the time of sample collection an important factor to consider. Additionally, a quenching step should also be implemented in the workflow to prevent metabolite turnover while preserving the concentration of metabolites at their physiological state (112–114). Additionally, certain metabolites, such as glutathione, require careful stabilization.

Biological variability and confounding factors require careful assessment in metabolomic studies. Because the metabolome is highly sensitive to both genetic and environmental influences, substantial variation can arise, which is unrelated to the biological process under investigation. In host-associated microbiology studies, factors including diet, lifestyle, medication use, disease status, sex, age, host genetics, microbiota composition, and interactions among these variables can profoundly influence metabolic profiles, complicating the identification of biologically meaningful changes. Consequently, rigorous control of confounding variables is essential for experimental design and data interpretation. In animal studies, biological variability can be minimized by using genetically similar animals housed under standardized environmental conditions. In contrast, controlling these variables in clinical studies is considerably more challenging. Therefore, careful cohort selection, strict inclusion and exclusion criteria (e.g., age, body mass index, disease status, and medication use), larger sample sizes (power analysis), and standardized protocols for diet and sample collection are often required to reduce interindividual variability. To promote reproducibility and facilitate experimental design, the Metabolomics Workbench, originally established through the NIH Common Fund Metabolomics Program, provides a valuable community resource that includes standardized protocols, study design recommendations, training materials, and publicly available metabolomics data sets for method development and benchmarking (115).

Next, biological variability also extends to microbial systems. Even genetically identical microorganisms can exhibit substantial metabolic heterogeneity because of differences in growth phase, nutrient availability, oxygen gradients, cell density, and stochastic gene expression. In polymicrobial communities, metabolite production is further shaped by interspecies interactions, competition, and cross-feeding, making metabolic profiles highly dependent on community composition and environmental context. Consequently, careful experimental standardization and reporting, including inoculum size, culture conditions, growth stage, and sampling time, is essential to ensure reproducible metabolomic measurements.

Finally, metabolomic data analysis remains one of the major challenges in the field. A typical untargeted metabolomics data analysis workflow involves feature detection (like generation of OTU or ASV table for microbiome analysis), feature alignment, normalization, statistical analysis, and structural annotation of metabolites (99, 100). Among these steps, structural annotation is widely regarded as the principal bottleneck, with only approximately 10% of detected features receiving confident structural annotations in a typical untargeted data set (116). This low annotation rate reflects the extraordinary chemical diversity of metabolites, the limited availability of authentic standards, incomplete spectral databases, and the often-unpredictable fragmentation behavior of structurally diverse molecules. These challenges are particularly pronounced in microbial data sets, where many specialized metabolites remain undiscovered and are therefore absent from existing reference libraries.

Current annotation strategies rely on multiple complementary approaches, including matching MS/MS spectra to public spectral libraries (117–120), comparison with authentic chemical standards, and computational prediction of fragmentation patterns and molecular structures using *in silico* tools (121–125). Molecular networking has further expanded annotation capabilities by organizing metabolites according to MS/MS spectral similarity, allowing structural information from annotated compounds to be propagated to related analogs and facilitating the discovery of new metabolite families (117, 126–128). Despite these advances, most metabolite annotations remain putative, highlighting the continued need for improved computational methods, expanded community spectral libraries, and systematic characterization of microbial metabolites. Finally, absolute quantification of metabolites necessitates the use of standards (which are often not available for specialized metabolites), given the differences in metabolite ionization efficiencies.

## CONCLUSION

Metabolomics has emerged as a powerful approach for characterizing the molecular phenotype of microorganisms and their interactions with drugs, one another, their hosts, and the surrounding environment. By providing a direct readout of biochemical activity, metabolomics complements other omics technologies and has enabled new insights into microbial physiology, metabolism, chemical communication, biotransformation, and natural product biosynthesis. Although challenges related to metabolome coverage, experimental design, metabolite annotation, and data interpretation remain, continued advances in analytical instrumentation, user-friendly computational methods, and community resources are steadily improving the accessibility and rigor of metabolomics.

Looking ahead, innovations in metabolite annotation, spatial metabolomics, artificial intelligence-assisted data analysis, and multi-omics integration promise to further transform microbiological research. Together, these advances will enable more comprehensive investigations of microbial physiology, ecological interactions, and host-microbe communication while accelerating biomarker discovery, natural product research, microbiome engineering, and the development of microbiome-based therapeutics.
